# Harrowing experiences of vagina fistula victims in the Kpandai District of Ghana: A qualitative study

**DOI:** 10.1371/journal.pone.0362368

**Published:** 2026-11-06

**Authors:** Eric Elorm Ahorsu, Joshua Adeti, Ernest Kemevor Selasie, Mohammed Ibn Shamsudeen, Cynthia Akos Okumasi, Benjamin Amedume, Justice Wiston Amstrong Jonathan

**Affiliations:** 1 Department of Nursing, School of Nursing and Midwifery, University of Health and Allied Sciences, Ho, Ghana; 2 Department of Health Information, Ho Teaching Hospital, Ho, Ghana; 3 Department of Basic Sciences, School of Basic and Biomedical Sciences, University of Health and Allied Sciences, Ho, Ghana; Biology and Health Laboratory, Ibn Tofail University, MOROCCO

## Abstract

**Background:**

Vaginal fistula (VF), particularly vesicovaginal fistula (VVF), remains a significant reproductive health challenge in many low-resource settings, including the Kpandai District of Ghana. Despite its profound physical and psychosocial impact, the lived experiences of affected women in rural districts remain under-documented.

**Aim:**

This study explored the lived experiences of women diagnosed with vesicovaginal fistula in the Kpandai District of Ghana, focusing on their physical, emotional, and social challenges, as well as coping strategies.

**Methods:**

A qualitative descriptive study was conducted using in-depth interviews with ten women with clinically confirmed VVF. Participants were initially identified through a district health facility and subsequently recruited using a combination of purposive and snowball sampling techniques. Interviews were audio-recorded, transcribed verbatim, and analysed thematically.

**Results:**

Prolonged and obstructed labour—often occurring at home—emerged as the primary reported cause of VVF. Cultural beliefs, gender-based decision-making dynamics, limited access to healthcare, and financial constraints were key barriers to timely medical intervention. Consequences included persistent urinary incontinence, social stigma, emotional trauma, marital dissolution (reported by eight participants), loss of livelihood, and social isolation. Coping strategies included frequent washing, use of absorbent materials, and reliance on perfumes or herbal remedies.

**Conclusions:**

Vesicovaginal fistula remains a devastating yet largely preventable condition in rural Ghana. The findings highlight the need to strengthen maternal health services, improve timely referral systems, and integrate psychosocial support within community-based care frameworks.

## Introduction

Vaginal fistula (VF), particularly obstetric fistula (OF), is a debilitating childbirth-related injury characterized by an abnormal communication between the vaginal canal and adjacent organs such as the bladder (vesicovaginal fistula), rectum (rectovaginal fistula), or urethra (urethrovaginal fistula). These pathological connections lead to continuous involuntary leakage of urine or faeces, resulting in profound physical, psychological, and social consequences [1–4]. Although fistulas may also result from pelvic surgeries, malignancies, or radiation therapy, the leading cause in low- and middle-income countries (LMICs) is prolonged and obstructed labour in the absence of timely and skilled obstetric care [5–7].

The clinical manifestations of VF vary depending on the type and location. Vesicovaginal fistula (VVF), the most common form, causes continuous urinary incontinence and can lead to chronic infections and excoriation of the skin [8]. Rectovaginal fistula (RVF) results in faecal incontinence and recurrent urinary tract or vaginal infections [9,10]. Urethrovaginal fistula (UVF) and other less common types, such as cervicovaginal and enterovaginal fistulas, also significantly impair the quality of life, sexual function, and reproductive health of affected women [11–13]. These physical symptoms are often compounded by stigma, ostracization, depression, and socioeconomic marginalization [14,15].

Globally, the burden of VF has declined in high-income countries due to advances in maternal health, obstetric surgical techniques, and timely emergency obstetric interventions. In contrast, it remains a serious public health concern in parts of sub-Saharan Africa, South Asia, and the Middle East [2,16]. In these regions, VF is a stark indicator of systemic failures in maternal health systems. Conservative estimates suggest that over two million women, primarily in LMICs, are living with untreated obstetric fistulas, with thousands of new cases occurring annually [17–19]. Most affected women reside in impoverished rural communities where early marriage, adolescent pregnancy, female illiteracy, malnutrition, and harmful cultural practices are prevalent risk factors [20–22].

In West Africa, the incidence of VF is particularly high. Countries such as Nigeria, Niger, Sierra Leone, and Guinea account for a substantial proportion of global cases [23,24]. Nigeria alone is estimated to have over 150,000 untreated cases, with new cases outpacing surgical repairs due to insufficient resources and infrastructural gaps [20,25]. While efforts by organizations such as UNFPA, the Fistula Foundation, and regional governments have made commendable progress in improving surgical repair rates and raising awareness, comprehensive care – including mental health services, community reintegration programs, and preventive strategies – remains inadequate in many settings [26–28].

Ghana faces similar challenges, particularly in rural and underserved districts such as Kpandai in the Northern Region, where health infrastructure is limited and sociocultural barriers hinder access to maternal healthcare. Factors such as entrenched gender roles that limit women’s autonomy in seeking care, traditional beliefs attributing prolonged labour to spiritual or moral causes, and widespread poverty contribute to delays in obtaining skilled obstetric services [29–31]. Although Ghana has implemented national strategies aimed at reducing maternal morbidity – including the Community-based Health Planning and Services (CHPS) initiative and the National Health Insurance Scheme (NHIS) – gaps persist in the identification, treatment, and long-term support of women with VF.

While previous research has largely focused on the clinical and epidemiological dimensions of VF, limited attention has been given to the lived experiences of affected women in rural Ghana. Understanding these experiences is essential for contextualizing the multifaceted impacts of the condition, from the individual to the community level, and for designing culturally responsive interventions that address both medical and social dimensions of care.

This qualitative study therefore seeks to explore the lived experiences of women affected by vaginal fistula in the Kpandai District of Ghana. It aims to illuminate the physical, emotional, social, and economic burdens endured by these women, the barriers they face in accessing care, and the coping strategies they employ. The findings are expected to contribute to evidence-informed policymaking, enhance understanding of the social determinants of VF, and support national and regional efforts to eliminate obstetric fistulas through prevention, treatment, and rehabilitation.

## Materials and methods

### Study design and setting

A qualitative descriptive design was adopted to explore the lived experiences of women living with vesicovaginal fistula in the Kpandai District of Northern Ghana.

Kpandai District is predominantly rural and was carved out of the former East Gonja District in 2008. It has an estimated population of approximately 108,816, with the majority residing in rural communities. The district is characterized by limited infrastructure, subsistence farming as the dominant livelihood, and significant geographic barriers that affect access to healthcare services.

The district’s healthcare infrastructure consists mainly of health centres and Community-based Health Planning and Services (CHPS) compounds. Basic maternity services are available at selected facilities; however, there were no resident gynaecologists at the Kpandai District Hospital during the study period. Participants reported initially seeking care at Buya Health Centre, where they were attended to by the midwife in-charge and received maternal care. Suspected fistula cases were referred to the Kpandai District Hospital and subsequently to the Tamale Fistula Centre for specialist management. The absence of resident specialist care within the district represents a significant barrier to timely diagnosis and treatment.

### Study population and sampling

Participants were initially identified through records and referrals from the Kpandai District Health Directorate and selected healthcare facilities within the district. The midwife in-charge at Buya Health Centre provided background information regarding known cases and assisted in locating initial participants.

A combination of purposive and snowball sampling techniques was employed. After initial participants were identified through the health facility, they subsequently assisted in identifying and referring other eligible women living with vesicovaginal fistula within the district.

Clinical records confirmed that all participants were diagnosed with vesicovaginal fistula (VVF). Diagnosis was based on documented health facility records and corroborated by participants’ self-reports and obstetric histories obtained during interviews. A total of ten women who met the inclusion criteria consented to participate in the study

### Data collection tool

Interview format was employed in the data collection. Structured in-depth interviews were conducted using an interview guide (S1 Data) comprising 25 open-ended questions grouped into four thematic areas: socio-demographics, causes of VF, psychosocial impact, and coping strategies. All interviews were audio-recorded, transcribed verbatim, and supplemented with observational notes. Verbal informed consent was obtained from all participants before they participated in the study. Data were collected between August 16, 2018 and December 15, 2018 after ethical clearance was obtained.

### Ethical considerations

Ethical approval for this study was obtained from the University of Health and Allied Sciences Research Ethics Committee (UHAS-REC A.10 [8] 17–18).

Verbal informed consent was obtained from all participants. This approach was adopted due to the low literacy levels among participants and the community-based snowball recruitment strategy employed in the study. Participants were fully informed about the purpose of the study, procedures involved, potential risks and benefits, and their right to withdraw at any time without consequences. Confidentiality and anonymity were strictly maintained.

### Data analysis

Audio-recorded interviews were transcribed verbatim and reviewed for accuracy. Data were analysed using thematic analysis following the approach described by Braun and Clarke (2006). The analysis involved familiarization with the data through repeated reading of transcripts, generation of initial codes, identification of patterns across participants, and development of overarching themes. Coding and theme development were conducted collaboratively among members of the research team to enhance credibility and reduce interpretive bias. Discrepancies were discussed and resolved through consensus.

Illustrative quotations were selected to represent the range and depth of participants’ perspectives.

## Results

### Demographic characteristics of participants

The demographic characteristics of participants (**Table 1**) were diverse in some respects, whereas others were similar. The participants aged from 16 to 70 years. However, some ages were approximate because several participants did not know their exact dates of birth and estimated their ages based on recalled life events. Although participants belonged to different ethnic groups, they all spoke Likpakpa. Most had no formal education and engaged in informal occupations such as farming and petty trading. All participants reported that the onset of VVF followed prolonged labour at home.

**Table 1:** Sociodemographic Profile of Participants.

Participants	Age	Present marital status	Previous Employment	Years of fistula experience	Parity
P1	45	Married	Petty trading	7	G4P2A1
P2	43	Divorced	Peasant farming	6	G4P3A1
P3	70	Divorced	Peasant farming	15	G7P6A1
P4	39	Separated	Petty trading	5	G6P4+1D
P5	43	Divorced	Soap making	7	G5P4A1
P6	16	Married	Peasant farming	2	G2P1
P7	22	Divorced	Hair dressing	4	G2P1A
P8	30	Separated	Petty trading	5	G3P2
P9	35	Divorced	Porridge selling	6	G3P2A1
P10	40	Divorced	Petty Trading	7	G9P4

Analysis of the participants’ narratives revealed five major themes shown in Table 2.

**Table 2:** Themes.

THEMES	HEADINGS
Theme 1	Sociocultural and Health System Factors Contributing to Vaginal Fistula
Theme 2	Psychosocial and Emotional Distress
Theme 3	Stigma and Social Exclusion
Theme 4	Marital Breakdown and Economic Disempowerment
Theme 5	Coping Strategies and Sources of Resilience

These themes capture the multifaceted burden of living with vaginal fistula in the Kpandai District and are presented below with illustrative participant quotes

#### Theme 1: Sociocultural and Health System Factors Contributing to Vaginal Fistula.

Participants commonly reported prolonged and obstructed labour as the primary antecedent to their condition, often exacerbated by delayed decision-making, reliance on traditional birth attendants, and limited access to emergency obstetric services.

Male dominance in household decisions and prevailing cultural beliefs contributed significantly to these delays.

*“My husband didn’t allow me to go to the hospital. He said the elders were praying and it would be fine. I was in labour at home for three days before they agreed to take me.”* (Participant 4)

*“The road to the clinic is very bad, and we had no money for a car. So I stayed at home until things got worse.”* (Participant 7)

Several women attributed their condition to misconceptions about spiritual causes, which influenced health-seeking behaviour.

*“They said I had been cursed or did something wrong, so they took me to a shrine first. By the time I reached the hospital, I had already started leaking.”* (Participant 1)

#### Theme 2: Psychosocial and Emotional Distress.

The constant leakage of urine led to intense feelings of shame, despair, and social withdrawal

Many participants expressed a deep sense of loss of identity and dignity.

*“I no longer feel like a woman. I can’t control myself, and I smell. It’s like I’ve become invisible.”* (Participant 3)

*“At night, I cry quietly. I ask God why this happened to me. Sometimes, I wish I wouldn’t wake up.”* (Participant 5)

The psychological toll was often compounded by self-blame and hopelessness, especially for women who had lived with the condition for several years.

*“I thought I had failed my husband, failed as a mother. I felt useless.”* (Participant 8)

#### Theme 3: Stigma and Social Exclusion.

Many participants experienced severe stigmatization, both within their families and communities. The persistent odour and visible leakage led to exclusion from religious, social, and communal activities.

*“People in church avoid sitting near me. Some cover their noses. It’s humiliating.”* (Participant 6)

*“Even my own relatives won’t let me sleep in their rooms. They say I bring shame to the family.”* (Participant 10)

This rejection extended to public spaces and even healthcare settings.

*“When I went to the clinic for another issue, the nurse made a face and told me to stand outside. I didn’t go back.”* (Participant 9)

#### Theme 4: Marital Breakdown and Economic Disempowerment.

The social and physical consequences of VF often led to marital instability or divorce, and many women reported being abandoned or replaced by their husbands.

*“After I started leaking, my husband said he couldn’t stand it. He brought in another wife and sent me to my parents.”* (Participant 3)

*“I tried to be intimate with him, but he said I disgusted him. I haven't seen him in years.”* (Participant 4)

Of the ten participants, eight reported marital dissolution following the onset of vesicovaginal fistula, while two continued to live in their marital homes. Those who experienced separation explicitly attributed it to the condition, reporting that their partners cited persistent urinary leakage and associated odour as reasons for abandonment or remarriage. The condition also had a direct impact on economic productivity. Women were unable to work, farm, or trade, leading to loss of income and dependency.

*“I used to sell food at the market, but no one would buy from me because of the smell. Now I just stay home.”* (Participant 2)

#### Theme 5: Coping Strategies and Sources of Resilience.

Despite their challenges, participants demonstrated remarkable resilience, adopting practical and spiritual coping mechanisms. Many used absorbent materials, reduced public interaction, or developed strict hygiene routines.

*“I wear layers of cloth and change often. I clean myself many times a day so people won’t notice.”* (Participant 8)

Others drew strength from religious faith, peer encouragement, or the hope of surgery.

*“I haven’t lost hope. I pray every day and believe God will send someone to help me.”* (Participant 5)

*“Meeting another woman with the same problem gave me courage. I realized I’m not cursed, just unlucky.”* (Participant 1)

### Summary of findings

The voices of the participants revealed that vaginal fistula is not merely a medical condition, but a deeply stigmatizing, isolating, and life-altering experience. The intersection of gender norms, cultural beliefs, systemic health barriers, and poverty shapes both the causes and consequences of VF. Yet, amidst the suffering, women displayed extraordinary resilience, offering a powerful call for responsive and compassionate health systems.

## Discussion

This study explored the lived experiences of women affected by vaginal fistula (VF) in the Kpandai District of Ghana, revealing harrowing physical, psychological, social, and economic consequences. The findings confirm that VF is not merely a medical complication, but a multifaceted condition deeply rooted in sociocultural and systemic factors, reflecting broader inequities in maternal healthcare in low-resource settings.

Socially, VF victims face stigmatization, isolation, and exclusion, consistent with existing literature [32–34]. Emotionally, the distress of abandonment and public humiliation has lasting psychological effects [35,36]. Economically, the inability to work or maintain businesses due to incontinence and social stigma exacerbates poverty among affected women.

Consistent with previous studies across sub-Saharan Africa (Ngongo et al., 2019; Swain et al., 2020), participants in this study attributed the development of fistulas primarily to prolonged and obstructed labour, often worsened by delayed access to emergency obstetric care. These delays were largely the result of entrenched patriarchal norms, where decision-making power rests with male family members, as well as economic constraints and geographic inaccessibility of healthcare facilities. These findings corroborate the “three delays” model – delays in deciding to seek care, reaching care, and receiving adequate care – which continues to be a central barrier to safe delivery in many rural communities [37].

The sociocultural context surrounding childbirth in Kpandai further complicates timely access to care. Participants reported that cultural beliefs associating prolonged labour with spiritual punishment or infidelity led to the marginalization of affected women. Similar beliefs have been documented in other rural African settings, where VF is often seen as a manifestation of divine retribution or as evidence of personal failure [14,34]. Such stigmatizing interpretations not only delay treatment but also deepen the psychological trauma experienced by victims.

The psychosocial impact of VF in this study was profound. Women reported feelings of shame, loss of dignity, and isolation due to persistent urinary l incontinence [33,36]. These narratives mirror findings from other qualitative studies across Nigeria, Ethiopia, and Uganda, where women with VF are frequently divorced, excluded from communal activities, or abandoned by family members (Morhason-Bello et al., 2020; Maheu-Giroux et al., 2015). The persistent odour associated with incontinence made social reintegration difficult, and many participants described being treated as outcasts or cursed individuals.

Economically, the condition rendered many women unemployable or unable to continue income-generating activities such as farming or trading, due to physical discomfort, stigma, or social avoidance. This cycle of poverty and exclusion has been emphasized in prior research showing that VF not only results from poverty but also perpetuates it (Cowgill et al., 2015; Bello et al., 2020). In the absence of comprehensive rehabilitation programs, affected women remain marginalized and dependent.

Despite these overwhelming challenges, the study also uncovered important coping strategies employed by the women. Some relied on religious faith, others adopted physical strategies such as wearing absorbent materials or avoiding public gatherings. These responses, while indicative of resilience, also highlight the lack of structured psychosocial support or community-based rehabilitation initiatives in the district. This aligns with previous calls for a holistic model of fistula care that extends beyond surgical repair to include counselling, skills training, and social reintegration [2,18].

The findings further emphasize that while surgical intervention is critical, it is insufficient in addressing the long-term consequences of VF. Reconstructive surgery is often inaccessible to women in remote settings like Kpandai due to financial constraints, poor health infrastructure, and lack of specialized personnel. Moreover, several participants had lived with VF for years without knowing that surgical treatment was available—an indication of inadequate health education and outreach. This reinforces WHO’s recommendation that fistula prevention and management should be integrated into broader maternal health policies, especially within primary healthcare systems [2].

Although no structured district-wide fistula rehabilitation program existed at the time of the study, the midwife at Buya Health Centre collaborated with local non-governmental organizations to provide limited support to affected women, including toiletries and small financial assistance. However, these efforts were informal and not part of a sustained or structured rehabilitation framework.

This study contributes to the limited but growing body of evidence highlighting the intersection of gender, poverty, culture, and health system failures in shaping VF outcomes in Ghana. It provides critical qualitative insight into the real-world impact of obstetric fistula and underscores the need for multisectoral strategies to eliminate it. These should include strengthening maternal health services, enhancing community awareness, empowering women to make reproductive health decisions, and investing in post-repair reintegration programs.

### Strengths and limitations

This study has several notable strengths. First, it provides in-depth qualitative insights into the lived experiences of women with vaginal fistula in a rural and under-studied district of Ghana, a context that is rarely represented in the literature. By centering participants’ narratives, the study captures the complex physical, psychosocial, and economic dimensions of VVF that are often overlooked in clinical or epidemiological research. The use of in-depth interviews allowed participants to articulate their experiences in their own words, enhancing the richness and credibility of the findings. Initial recruitment through health facility records and referrals ensured that all participants had clinically confirmed diagnoses.

However, certain limitations should be acknowledged. The small sample size and focus on a single rural district limit the transferability of findings to other contexts. Reliance on self-reported obstetric histories may introduce recall bias, particularly among women who had lived with the condition for several years. Additionally, the absence of comprehensive district-level data on specialized fistula services limited the ability to quantitatively assess health system capacity, although this gap itself highlights systemic weaknesses in service provision.

### Implications for nursing practice and health policy

The findings of this study highlight the need for a comprehensive and context-sensitive response to vaginal fistula in rural Ghana. Given that prolonged and obstructed labour emerged as the primary reported antecedent to VVF among participants, strengthening community-level maternal health education is essential. The capacity of nurses and community health workers, particularly within the Community-based Health Planning and Services (CHPS) framework, could be strengthened to provide targeted education on early recognition of prolonged labour, timely referral, and the importance of skilled birth attendance.

Improving access to skilled and emergency obstetric care in rural districts such as Kpandai is critical. Investment in rural health infrastructure, transportation systems, and the deployment of adequately trained midwives and obstetric personnel would reduce delays in seeking, reaching, and receiving care. Integrating fistula awareness and screening into routine maternal health services may also facilitate early identification and referral.

The psychosocial and economic consequences reported by participants underscore the importance of holistic nursing care that extends beyond surgical repair. Incorporating counselling services, peer-support groups, and post-repair reintegration programs into district health systems could help address stigma, restore dignity, and improve quality of life. Strengthening nursing education to include fistula prevention, management, and psychosocial rehabilitation would further enhance care delivery in low-resource settings.

At the policy level, efforts should focus on addressing sociocultural and gender-related barriers that delay healthcare-seeking behaviour. Community engagement strategies that involve male partners, traditional leaders, and religious institutions may help challenge harmful beliefs and promote shared decision-making in maternal health. Furthermore, sustained investment in fistula surveillance, prevention, and referral systems is necessary to ensure that women in remote districts are not left without access to specialized care.

The proposed implications are informed by participants’ lived experiences and expressed needs, observations of health system gaps within the district, and alignment with national and international maternal health frameworks, including WHO (2018) and UNFPA (2020) recommendations.

## Conclusions

This study provides in-depth insight into the lived experiences of women affected by vaginal fistula in the Kpandai District of Ghana, revealing that the condition extends far beyond a clinical complication to encompass profound psychological, social, and economic consequences. The findings demonstrate how delayed access to skilled obstetric care, entrenched gender norms, cultural beliefs, and health system limitations interact to shape both the occurrence and long-term impact of vaginal fistula.

While vaginal fistula is largely preventable, the persistence of cases in rural districts underscores ongoing inequities in maternal healthcare access and service delivery. Addressing these inequities requires strengthening primary healthcare systems, improving emergency obstetric services, and ensuring that prevention, treatment, and rehabilitation efforts are integrated within broader maternal health strategies.

Ultimately, centering the voices of affected women highlights the urgent need for compassionate, context-responsive, and system-level interventions that restore dignity and reduce preventable suffering. Continued research and policy commitment are essential to advancing equitable maternal health outcomes in low-resource settings.

## Supporting information

S1 Data Collection Instrument. (PDF)
