# Supplementary material for: Harrowing experiences of vagina fistula victims in the Kpandai District of Ghana: A qualitative study
Source: PLoS One. 2026 Nov 6;21(11):e0362368. doi: 10.1371/journal.pone.0362368 (PMC13742871; doi:10.1371/journal.pone.0362368)
Supplement: Supplementary file 1 [file pone.0362368.s001.pdf]

HARROWING EXPERIENCES OF VAGINAL FISTULA VICTIMS IN THE KPANDAI DISTRICT OF GHANA: A QUALITATIVE STUDY

DATA COLLECTION INSTRUMENT

Structured Interview Guide

You are free to ask any questions about the study or seek clarification during the interview. Please respond honestly and to the best of your knowledge.

SECTION A: SOCIO-DEMOGRAPHIC DATA

1. What is your age?
[ ] 15–25 years [ ] 26–35 years [ ] 36–45 years [ ] 46 years and above

2. What is your marital status?
[ ] Single [ ] In a relationship [ ] Married [ ] Divorced

3. Do you work?
[ ] Yes [ ] No

4. What is your occupation?
[ ] Unemployed [ ] Farmer [ ] Trader/Businesswoman [ ] Civil Servant [ ] Housewife [ ] Other (please specify): ______________________

5. What is your highest level of education?
[ ] None [ ] Primary [ ] Secondary [ ] Technical/Vocational [ ] Tertiary

6. What is your religious affiliation?
[ ] Christian [ ] Muslim [ ] Traditionalist [ ] Other (please specify):
____

7. How many pregnancies have you had?
[ ] 1–2 [ ] 3–4 [ ] 5–6 [ ] 7 or more

8. How old were you when you first became pregnant?
[ ] 15–18 years [ ] 19–21 years [ ] 22–25 years [ ] 26–29 years [ ] 30–33 years [ ] 34 years and above

9. How many children do you have?
[ ] None [ ] 1–2 [ ] 3–4 [ ] 5–6 [ ] 7 or more

SECTION B: CAUSES OF VAGINAL FISTULA

10. What was the duration of your labour?

____

11. Where did you deliver?

____

12. Who attended to your delivery?

____

SECTION C: EMOTIONAL, SOCIAL, PSYCHOLOGICAL, AND CULTURAL IMPACTS

13. How has your condition affected your social life or standing in the community?

____

14. How has your marital relationship changed since the experience?

____

15. How has your family responded to your condition?

____

16. How has your employment status been affected by the condition?

____

17. How were you treated by the society or people around you?

____

18. How has your condition affected your ability to work?

____

SECTION D: COPING STRATEGIES OF VAGINAL FISTULA VICTIMS

19. How do you relate to others now?

____

20. How often do you consume alcohol (if at all)?

____

21. How do you react to people's comments about you?

____

22. Have you sought any alternative treatment?

[ ] Yes [ ] No

If yes, where?

[ ] Herbalist [ ] Diviner [ ] Spiritualist [ ] Other: _______________________

23. How do you protect your clothing from soiling?

____

24. How do you support yourself?

[ ] With the help of relatives [ ] With the help of my husband [ ] Other (please specify): _______________________

25. How do you manage or reduce the odour?

[ ] With strong perfume [ ] With frequent bathing [ ] Other (please specify): _______________________

26. How often do you seek medical treatment?

____
