# Supplementary material for: Harrowing experiences of vagina fistula victims in the Kpandai District of Ghana: A qualitative study
Source: PLoS One. 2026 Nov 6;21(11):e0362368. doi: 10.1371/journal.pone.0362368 (PMC13742871; doi:10.1371/journal.pone.0362368)
Supplement: Supplementary file 2 [file pone.0362368.s003.pdf]

Response to Editorial Request – Data Availability Contact Information

Dear Ms. Ramos,

Thank you for your message and for the opportunity to revise our submission.
In response to your request, we have now provided a non-author institutional contact for the University of Health and Allied Sciences Research Ethics Committee (UHAS-REC) in the Data Availability Statement.

Contact details are as follows:
University of Health and Allied Sciences Research Ethics Committee (UHAS-REC)
Email: ihr@uhas.edu.gh
The manuscript has been updated accordingly, and the revised files have been uploaded.

Thank you for your guidance and support.
Kind regards,

Dr. Justice Wiston Amstrong Jonathan
