# Supplementary material for: Harrowing experiences of vagina fistula victims in the Kpandai District of Ghana: A qualitative study
Source: PLoS One. 2026 Nov 6;21(11):e0362368. doi: 10.1371/journal.pone.0362368 (PMC13742871; doi:10.1371/journal.pone.0362368)
Supplement: Supplementary file 3 [file pone.0362368.s004.pdf]

Journal: PLOS ONE

Manuscript ID: [PONE-D-25-43446R1] - [EMID:a9794f157f91879a]

Manuscript Title: Harrowing experiences of vagina fistula victims in the Kpandai District of Ghana: A qualitative study

Authors: Eric Elorm Ahorsu[1], Adeti Joshua[1], Ernest Kemevor Selasie[1], Mohammed Ibn Shamsudeen[1], Cynthia Akos Okumasi[1], Benjamin Amedume[3], Justice Wiston Amstrong Jonathan[2]

Dear Editor,

POINT-BY-POINT RESPONSE TO ACADEMIC EDITOR AND REVIEWER # 2 COMMENTS

We sincerely thank the Academic Editor and Reviewer #2 for the careful assessment of our revised manuscript and for acknowledging that the major methodological and reporting concerns raised in the previous round have been satisfactorily addressed. We have carefully considered the remaining comments and made the requested minor revisions. Our point-by-point responses and the corresponding changes made to the manuscript are provided below.

S/No.	Editorial/Reviewer #2 Comments	Authors' Response	Page & Line
	**Journal Requirements**		
1.	If the reviewer comments include a recommendation to cite specific previously published works, please review and evaluate these publications to determine whether they are relevant and should be cited.	Thank you. Reviewer #2 did not recommend any specific publications for citation in the current review round. Accordingly, no citation changes were required in response to this requirement.	N/A
2.	Please review your reference list to ensure that it is complete and correct. If you have cited papers that have been retracted, please include the rationale for doing so in the manuscript text, or remove these references and replace them with relevant current references.	Thank you. We have carefully reviewed the reference list for completeness, accuracy, consistency, and current publication status. No intentionally cited retracted articles were identified. Minor corrections to bibliographic details and reference formatting were made where necessary, including replacement/removal of inappropriate third-party URLs and use of DOI or	Page 22 Lines 458-459; page 25 lines 526-527; 530-531

		legitimate source information where available.	
3.	**Additional Editorial Comments**		
	Based on the reviewer's report and our assessment, the manuscript still requires further minor revision before it can be considered for publication. The authors should comprehensively address the remaining methodological and interpretive concerns and ensure that all requested changes are clearly incorporated into the manuscript.	Thank you for the opportunity to further improve the manuscript. We have carefully addressed the remaining methodological and interpretive issues identified by Reviewer #2. Specifically, we have ensured consistency regarding the verbal informed-consent procedure and refined statements concerning prolonged and obstructed labour so that the evidentiary basis for this finding is accurately represented. We also conducted a final consistency check throughout the manuscript.	
	**Reviewer #2 Comments**		
1.	The manuscript states that verbal informed consent was obtained, whereas the submission ethics statement appears to mention "written informed consent". This should be corrected for consistency.	We thank the reviewer for identifying this inconsistency. We confirm that **verbal informed consent**, rather than written informed consent, was obtained from all participants. The Ethical Considerations subsection of the submitted manuscript already correctly stated that verbal informed consent was obtained. The reference to written informed consent in the submission ethics statement was an inadvertent error and has now been corrected to accurately reflect the consent procedure used in the study. For additional consistency within the manuscript, we have also revised the Data Collection	

		Tool subsection to explicitly specify "verbal informed consent."<br><br>**Revision made in the manuscript – Data Collection Tool subsection:**<br>Previous wording:<br>"Informed consent was obtained from all participants before they participated in the study."<br>**Revised wording:**<br>"Verbal informed consent was obtained from all participants before they participated in the study."<br>The Ethical Considerations subsection continues to state: "Verbal informed consent was obtained from all participants. This approach was adopted due to the low literacy levels among participants and the community-based snowball recruitment strategy employed in the study."<br>The submission ethics statement has also been corrected accordingly.	Page 7 Line 143
2.	Also, because the attribution of prolonged labour appears to rely partly on participant narratives and obstetric histories, the authors should consistently describe this as the "reported" or "documented/reported" cause where appropriate.	We thank the reviewer for this important clarification. We agree that statements concerning the relationship between prolonged and obstructed labour and VVF should accurately reflect the evidentiary basis of the study and should not imply independent clinical determination of causality where	

		this was based on participants' accounts and obstetric histories. We note that the Abstract of the submitted manuscript already described prolonged and obstructed labour as the "primary reported cause of VVF," and the Discussion similarly attributed this finding explicitly to participants' accounts. We have retained these appropriately qualified statements.<br>We have, however, identified and revised the remaining statements in the Results and Implications sections where the wording could be interpreted as asserting causality more directly.<br><br>**Revisions made in the manuscript:**<br>**Results – Demographic Characteristics of Participants**<br>Previous wording:<br>"All reported onset of VF following prolonged labour at home."<br>**Revised wording:**<br>"All participants reported that the onset of VVF followed prolonged labour at home."<br><br>**Results – Theme 1: Sociocultural and Health System Factors Contributing to Vaginal Fistula**<br>Previous wording:<br>"Participants commonly described prolonged and	Page 8/9 Lines 177-178

		obstructed labour as the primary cause of their condition, often exacerbated by delayed decision-making, reliance on traditional birth attendants, and limited access to emergency obstetric services."<br>**Revised wording:**<br>"Participants commonly reported prolonged and obstructed labour as the primary antecedent to their condition, often exacerbated by delayed decision-making, reliance on traditional birth attendants, and limited access to emergency obstetric services."<br><br>**Implications for Nursing Practice and Health Policy**<br>Previous wording:<br>"Given that prolonged and obstructed labour emerged as a primary cause, strengthening community-level maternal health education is essential."<br>**Revised wording:**<br>"Given that prolonged and obstructed labour emerged as the primary reported antecedent to VVF among participants, strengthening community-level maternal health education is essential."<br>These revisions ensure that the manuscript consistently distinguishes participants' reported obstetric histories from independently established clinical causation.	Page 10 Lines 192-194<br><br>Pages 17 Lines 348-350

1.	**Optional Revisions Made**	**Revisions Made**	**Page & Line**
	The authors also carried out optional/general manuscript audit to ensure internal consistency.	These essentially included correction of typographical errors, language polishing and minor formatting errors.	Page 7 Line 143; Page 8 Lines 170-176; Page 10 line 209; Page 14 Line 292; Page 17 Lines 351-353
