# Supplementary material for: Harrowing experiences of vagina fistula victims in the Kpandai District of Ghana: A qualitative study
Source: PLoS One. 2026 Nov 6;21(11):e0362368. doi: 10.1371/journal.pone.0362368 (PMC13742871; doi:10.1371/journal.pone.0362368)
Supplement: Peer review history

# Peer review: Decision Letter 0

**DOI:** 10.1371/journal.pone.0362368.r001

**Authors:** Miloud Chakit

5 Feb 2026

Dear Dr. JONATHAN,

Please submit your revised manuscript by Mar 22 2026 11:59PM. If you will need more time than this to complete your revisions, please reply to this message or contact the journal office at plosone@plos.org

- A letter that responds to each point raised by the academic editor and reviewer(s). You should upload this letter as a separate file labeled 'Response to Reviewers'.
- A marked-up copy of your manuscript that highlights changes made to the original version. You should upload this as a separate file labeled 'Revised Manuscript with Track Changes'.
- An unmarked version of your revised paper without tracked changes. You should upload this as a separate file labeled 'Manuscript'.

If you would like to make changes to your financial disclosure, please include your updated statement in your cover letter. Guidelines for resubmitting your figure files are available below the reviewer comments at the end of this letter.

If applicable, we recommend that you deposit your laboratory protocols in protocols.io to enhance the reproducibility of your results. Protocols.io assigns your protocol its own identifier (DOI) so that it can be cited independently in the future. For instructions see: https://journals.plos.org/plosone/s/submission-guidelines#loc-laboratory-protocols. Additionally, PLOS ONE offers an option for publishing peer-reviewed Lab Protocol articles, which describe protocols hosted on protocols.io. Read more information on sharing protocols at https://plos.org/protocols?utm_medium=editorial-email&utm_source=authorletters&utm_campaign=protocols.

We look forward to receiving your revised manuscript.

Kind regards,

Miloud Chakit, PhD

Academic Editor

PLOS One

Journal Requirements:

When submitting your revision, we need you to address these additional requirements.

1. Please ensure that your manuscript meets PLOS ONE's style requirements, including those for file naming. The PLOS ONE style templates can be found at

https://journals.plos.org/plosone/s/file?id=wjVg/PLOSOne_formatting_sample_main_body.pdf and

https://journals.plos.org/plosone/s/file?id=ba62/PLOSOne_formatting_sample_title_authors_affiliations.pdf

2. Please amend either the title on the online submission form (via Edit Submission) or the title in the manuscript so that they are identical.

3. If the reviewer comments include a recommendation to cite specific previously published works, please review and evaluate these publications to determine whether they are relevant and should be cited. There is no requirement to cite these works unless the editor has indicated otherwise.

[Note: HTML markup is below. Please do not edit.]

Reviewers' comments:

Reviewer's Responses to Questions

**Comments to the Author**

1. Is the manuscript technically sound, and do the data support the conclusions?

Reviewer #1: Yes

Reviewer #2: Yes

**********

2. Has the statistical analysis been performed appropriately and rigorously?

Reviewer #1: Yes

Reviewer #2: Yes

**********

3. Have the authors made all data underlying the findings in their manuscript fully available?

The PLOS Data policy

Reviewer #1: Yes

Reviewer #2: Yes

**********

4. Is the manuscript presented in an intelligible fashion and written in standard English?

Reviewer #1: Yes

Reviewer #2: Yes

**********

Reviewer #1: I have no additional comments for the authors at this time. The manuscript appears to meet PLOS One's ethical requirements, and I did not identify any concerns pertaining to dual publication, research ethics, or publication ethics.

Reviewer #2: The authors have presented a well-written qualitative manuscript aimed at assessing the lived experiences of women affected by vaginal fistula in the Kpandai District of Ghana. The topic addressed is highly relevant and pressing, particularly in low-resource settings where obstetric fistula remains a significant public health and social problem. The manuscript contributes valuable insights into the social, psychological, and cultural impacts of vaginal fistula and highlights the need for comprehensive support systems for affected women.

While the study is important and the manuscript is generally strong, several methodological and reporting issues require clarification to improve rigor, transparency, and interpretability of the findings.

Comments

1.Consent Procedure

The manuscript reports the use of verbal consent, but the rationale for this approach is not adequately explained. Please clarify whether verbal consent was chosen due to participants’ educational levels, cultural considerations, or local/institutional ethical committee requirements. Additionally, confirmation that this consent process was approved by a recognized ethics review board should be explicitly stated.

2.Participant Recruitment and Sampling

The process through which the ten participants were identified and recruited requires clearer description. Please specify whether participants were recruited through healthcare facilities, hospital records, local NGOs, community leaders, or other mechanisms. Transparent reporting of recruitment pathways is essential for assessing selection bias and transferability.

3.Type of Vaginal Fistula

The manuscript does not specify the type(s) of vaginal fistula experienced by participants. Given the differing clinical, social, and management implications of various fistula types, this information should be clearly reported.

4.Confirmation and Etiology of Vaginal Fistula

The authors indicate that only confirmed cases of vaginal fistula were included and that prolonged labour was implicated as the cause. However, it is unclear how this confirmation was established. Please clarify whether diagnosis and attribution to prolonged labour were based on hospital records, clinical examination, imaging, surgical documentation, or participant self-report.

5.Marital Outcomes and Attribution

Among participants who were divorced or separated, it is unclear whether all cited vaginal fistula as the primary reason for marital dissolution or whether other contributing factors were identified. Clarification is necessary to avoid over-attribution and to accurately reflect participants’ lived experiences.

6.Healthcare Infrastructure Context (Kpandai District)

While the manuscript provides extensive descriptive detail about the Kpandai District, some of this contextual information could be streamlined. Instead, the authors are encouraged to include more targeted and relevant details on the local healthcare infrastructure, particularly as it relates to fistula care. This may include:

a)The number and type of healthcare facilities in the district (e.g., hospitals, health centres).

b)Availability of maternity services.

c)Presence (or absence) of trained gynecologists or surgeons capable of diagnosing and managing vaginal fistula.

d)Availability of nurses or midwives with experience in fistula care or rehabilitation.

If such data are unavailable, this limitation should be explicitly acknowledged and discussed, as the absence of specialized services is itself a critical finding with implications for care access and referral systems.

7.Holistic Nursing and Support Programs

The discussion advocates for holistic nursing and support programs for women with vaginal fistula; however, it is unclear whether any such programs currently exist in the study district or in other districts within Ghana. Please clarify whether these recommendations are based on existing local initiatives, national programs, or identified gaps in services.

8.Source and Basis of Recommendations

The manuscript would benefit from clearer articulation of how the recommendations were derived. Specifically, please clarify whether the recommendations are:

a)Entirely proposed by the research team based on study findings,

b)Informed by participant suggestions,

c)Adapted from national guidelines or policies, or

d)Aligned with international frameworks (e.g., WHO or UNFPA recommendations).

Explicitly identifying the origin of these recommendations would enhance their credibility, reproducibility, and relevance for policymakers and practitioners.

**********

what does this mean?). If published, this will include your full peer review and any attached files.

If you choose “no”, your identity will remain anonymous but your review may still be made public.

**Do you want your identity to be public for this peer review?**  For information about this choice, including consent withdrawal, please see our Privacy Policy

Reviewer #1: **Yes:**  Rasheed Agboola

Reviewer #2: No

**********

[NOTE: If reviewer comments were submitted as an attachment file, they will be attached to this email and accessible via the submission site. Please log into your account, locate the manuscript record, and check for the action link "View Attachments". If this link does not appear, there are no attachment files.]

To ensure your figures meet our technical requirements, please review our figure guidelines: https://journals.plos.org/plosone/s/figures

You may also use PLOS’s free figure tool, NAAS, to help you prepare publication quality figures: https://journals.plos.org/plosone/s/figures#loc-tools-for-figure-preparation.

NAAS will assess whether your figures meet our technical requirements by comparing each figure against our figure specifications.

# Author comment: Author response to Decision Letter 1

**DOI:** 10.1371/journal.pone.0362368.r002

20 Mar 2026

Journal: PLOS ONE

Manuscript ID: [PONE-D-25-43446] - [EMID:a9794f157f91879a]

Manuscript Title: Harrowing experiences of vagina fistula victims in the Kpandai District

of Ghana: A qualitative study

Authors: Eric Elorm Ahorsu1, Adeti Joshua1, Ernest Kemevor Selasie1, Mohammed Ibn

Shamsudeen1, Cynthia Akos Okumasi1, Benjamin Amedume3, Justice Wiston

Amstrong Jonathan2

Dear Editor,

POINT-TO-POINT RESPONSE TO REVIEWERS’ COMMENTS

We thank the reviewer for their insightful and constructive comments, which have significantly improved the rigor, clarity, and transparency of our manuscript. All comments have been carefully addressed, and corresponding revisions have been made in the manuscript. Detailed responses are provided below.

S/No. Reviewer’s Comments Authors’ Response Line & page

1. Consent Procedure

The manuscript reports the use of verbal consent, but the rationale for this approach is not adequately explained. Please clarify whether verbal consent was chosen due to participants’ educational levels, cultural considerations, or local/institutional ethical committee requirements. Additionally, confirmation that this consent process was approved by a recognized ethics review board should be explicitly stated.

We appreciate this important observation.

The consent procedure has now been clarified in the Ethical Considerations subsection. Verbal informed consent was used due to low literacy levels among participants and was explicitly approved by the University of Health and Allied Sciences Research Ethics Committee (UHAS-REC).

Page 7; Line 158 - 163

2. Participant Recruitment and Sampling

The process through which the ten participants were identified and recruited requires clearer description. Please specify whether participants were recruited through healthcare facilities, hospital records, local NGOs, community leaders, or other mechanisms. Transparent reporting of recruitment pathways is essential for assessing selection bias and transferability.

We have revised the Study Population and Sampling section to clearly describe how participants were identified and recruited through the District Health Directorate and healthcare facilities.

Page 7; Line 140 - 144

3. Type of Vaginal Fistula

The manuscript does not specify the type(s) of vaginal fistula experienced by participants. Given the differing clinical, social, and management implications of various fistula types, this information should be clearly reported.

This information has now been added to the Study Population and Sampling and Results sections.

Page 7; Line 145 – 146

4. Confirmation and Etiology of Vaginal Fistula

The authors indicate that only confirmed cases of vaginal fistula were included and that prolonged labour was implicated as the cause. However, it is unclear how this confirmation was established. Please clarify whether diagnosis and attribution to prolonged labour were based on hospital records, clinical examination, imaging, surgical documentation, or participant self-report.

We have clarified that confirmation was based on documented clinical diagnoses from health facilities and corroborated by participant narratives.

Page 7; Line 147 – 149

5. Marital Outcomes and Attribution

Among participants who were divorced or separated, it is unclear whether all cited vaginal fistula as the primary reason for marital dissolution or whether other contributing factors were identified. Clarification is necessary to avoid over-attribution and to accurately reflect participants’ lived experiences.

Following careful re-examination of the data, we confirm that all participants who experienced marital dissolution explicitly attributed it to the onset of vesicovaginal fistula. The manuscript has been revised to clearly reflect this finding.

Page 13; Line 270-272

6. Healthcare Infrastructure Context (Kpandai District)

While the manuscript provides extensive descriptive detail about the Kpandai District, some of this contextual information could be streamlined. Instead, the authors are encouraged to include more targeted and relevant details on the local healthcare infrastructure, particularly as it relates to fistula care. This may include:

a)The number and type of healthcare facilities in the district (e.g., hospitals, health centres).

b)Availability of maternity services.

c)Presence (or absence) of trained gynaecologists or surgeons capable of diagnosing and managing vaginal fistula.

d)Availability of nurses or midwives with experience in fistula care or rehabilitation.

If such data are unavailable, this limitation should be explicitly acknowledged and discussed, as the absence of specialized services is itself a critical finding with implications for care access and referral systems.

The district description has been condensed, and targeted information on healthcare infrastructure relevant to fistula care has been added. Where data were unavailable, this has been explicitly acknowledged.

The district description has been condensed, and targeted information on healthcare infrastructure relevant to fistula care has been added. Where data were unavailable, this has been explicitly acknowledged.

Page 6; Line 124 – 131

7. Holistic Nursing and Support Programs

The discussion advocates for holistic nursing and support programs for women with vaginal fistula; however, it is unclear whether any such programs currently exist in the study district or in other districts within Ghana. Please clarify whether these recommendations are based on existing local initiatives, national programs, or identified gaps in services.

We thank the reviewer for this important observation. The manuscript has been revised to clarify that structured holistic nursing and psychosocial support programs specifically targeting women with vaginal fistula were not available in the Kpandai District at the time of the study. This clarification has been incorporated into the Discussion and the newly structured Implications for Nursing Practice and Health Policy section. The proposed interventions therefore reflect identified service gaps rather than existing local initiatives.

Page 18-19; Line 362 - 384

8. Source and Basis of Recommendations

The manuscript would benefit from clearer articulation of how the recommendations were derived. Specifically, please clarify whether the recommendations are:

a)Entirely proposed by the research team based on study findings,

b)Informed by participant suggestions,

c)Adapted from national guidelines or policies, or

d)Aligned with international frameworks (e.g., WHO or UNFPA recommendations).

Explicitly identifying the origin of these recommendations would enhance their credibility, reproducibility, and relevance for policymakers and practitioners. We appreciate this insightful comment. In the revised manuscript, the former “Recommendations” section has been integrated into a structured Implications for Nursing Practice and Health Policy section. We now explicitly state that the proposed implications are informed by: (i) participants’ lived experiences and expressed needs, (ii) gaps identified within the local health system during data collection, and (iii) alignment with national maternal health strategies and international guidelines, including WHO and UNFPA frameworks. This clarification enhances transparency regarding the evidentiary basis of the proposed interventions.

Final Note to Editor We believe these revisions have substantially strengthened the methodological transparency, contextual relevance, and interpretive accuracy of the manuscript. We are grateful to the reviewer for helping us improve the quality and clarity of this work.

Attachment Submitted filename: Response to Editorial Request.pdf

# Peer review: Decision Letter 1

**DOI:** 10.1371/journal.pone.0362368.r003

**Authors:** Miloud Chakit

26 Aug 2026

Dear Dr. JONATHAN,

Thank you for submitting your manuscript to PLOS One. After careful consideration, we feel that it has merit but does not fully meet PLOS One’s publication criteria as it currently stands. Therefore, we invite you to submit a revised version of the manuscript that addresses the points raised during the review process.

Please submit your revised manuscript by Oct 10 2026 11:59PM. If you will need more time than this to complete your revisions, please reply to this message or contact the journal office at plosone@plos.org. When you're ready to submit your revision, log on to https://www.editorialmanager.com/pone/ and select the 'Submissions Needing Revision' folder to locate your manuscript file.

- A letter that responds to each point raised by the academic editor and reviewer(s). You should upload this letter as a separate file labeled 'Response to Reviewers'.
- A marked-up copy of your manuscript that highlights changes made to the original version. You should upload this as a separate file labeled 'Revised Manuscript with Track Changes'.
- An unmarked version of your revised paper without tracked changes. You should upload this as a separate file labeled 'Manuscript'.

If you would like to make changes to your financial disclosure, please include your updated statement in your cover letter. Guidelines for resubmitting your figure files are available below the reviewer comments at the end of this letter.

If applicable, we recommend that you deposit your laboratory protocols in protocols.io to enhance the reproducibility of your results. Protocols.io assigns your protocol its own identifier (DOI) so that it can be cited independently in the future. For instructions see: https://journals.plos.org/plosone/s/submission-guidelines#loc-laboratory-protocols. Additionally, PLOS One offers an option for publishing peer-reviewed Lab Protocol articles, which describe protocols hosted on protocols.io. Read more information on sharing protocols at https://plos.org/protocols?utm_medium=editorial-email&utm_source=authorletters&utm_campaign=protocols.

As the corresponding author, your ORCID iD is verified in the submission system and will appear in the published article. PLOS supports the use of ORCID, and we encourage all coauthors to register for an ORCID iD and use it as well. Please encourage your coauthors to verify their ORCID iD within the submission system before final acceptance, as unverified ORCID iDs will not appear in the published article. *Only*  the individual author can complete the verification step; PLOS staff *cannot*  verify ORCID iDs on behalf of authors.

We look forward to receiving your revised manuscript.

Kind regards,

Miloud Chakit, PhD

Academic Editor

PLOS One

Journal Requirements:

1. If the reviewer comments include a recommendation to cite specific previously published works, please review and evaluate these publications to determine whether they are relevant and should be cited. There is no requirement to cite these works unless the editor has indicated otherwise.

2. Please review your reference list to ensure that it is complete and correct. If you have cited papers that have been retracted, please include the rationale for doing so in the manuscript text, or remove these references and replace them with relevant current references. Any changes to the reference list should be mentioned in the rebuttal letter that accompanies your revised manuscript. If you need to cite a retracted article, indicate the article’s retracted status in the References list and also include a citation and full reference for the retraction notice.

Additional Editor Comments:

Based on the reviewer’s report and our assessment, the manuscript still requires further minor revision before it can be considered for publication. The authors should comprehensively address the remaining methodological and interpretive concerns and ensure that all requested changes are clearly incorporated into the manuscript.

[Note: HTML markup is below. Please do not edit.]

Reviewers' comments:

Reviewer's Responses to Questions

**Comments to the Author**

Reviewer #2: All comments have been addressed

**********

2. Is the manuscript technically sound, and do the data support the conclusions?

Reviewer #2: Yes

**********

3. Has the statistical analysis been performed appropriately and rigorously?

Reviewer #2: Yes

**********

4. Have the authors made all data underlying the findings in their manuscript fully available?

The PLOS Data policy

Reviewer #2: Yes

**********

5. Is the manuscript presented in an intelligible fashion and written in standard English?

Reviewer #2: Yes

**********

Reviewer #2: The authors have made meaningful revisions and have addressed most of my prior concerns.

Only minor issues remain. First, the manuscript states that verbal informed consent was obtained, whereas the submission ethics statement appears to mention "written informed consent". This should be corrected for consistency.

Also, because the attribution of prolonged labour appears to rely partly on participant narratives and obstetric histories, the authors should consistently describe this as the “reported” or “documented/reported” cause where appropriate.

Overall, the authors have satisfactorily responded to the major methodological and reporting concerns, and only minor clarifications are needed.

**********

what does this mean?). If published, this will include your full peer review and any attached files.

If you choose “no”, your identity will remain anonymous but your review may still be made public.

**Do you want your identity to be public for this peer review?**  For information about this choice, including consent withdrawal, please see our Privacy Policy

Reviewer #2: No

**********

[NOTE: If reviewer comments were submitted as an attachment file, they will be attached to this email and accessible via the submission site. Please log into your account, locate the manuscript record, and check for the action link "View Attachments". If this link does not appear, there are no attachment files.]

To ensure your figures meet our technical requirements, please review our figure guidelines: https://journals.plos.org/plosone/s/figures

You may also use PLOS’s free figure tool, NAAS, to help you prepare publication quality figures: https://journals.plos.org/plosone/s/figures#loc-tools-for-figure-preparation.

NAAS will assess whether your figures meet our technical requirements by comparing each figure against our figure specifications.

# Author comment: Author response to Decision Letter 2

**DOI:** 10.1371/journal.pone.0362368.r004

2 Oct 2026

Response to editorial and reviewer # 2 comments have been submitted with the revised manuscript.

Attachment Submitted filename: RESPONSE TO REVIEWERS COMMENTS_R3.pdf

# Editor report: Decision Letter 2

**DOI:** 10.1371/journal.pone.0362368.r005

**Authors:** Miloud Chakit

25 Oct 2026

Harrowing experiences of vagina fistula victims in the Kpandai District of Ghana: A qualitative study

PONE-D-25-43446R2

Dear Dr. Jonathan,

We’re pleased to inform you that your manuscript has been judged scientifically suitable for publication and will be formally accepted for publication once it meets all outstanding technical requirements.

Within one week, you’ll receive an e-mail detailing the required amendments. When these have been addressed, you’ll receive a formal acceptance letter and your manuscript will be scheduled for publication.

An invoice will be generated when your article is formally accepted. Please note, if your institution has a publishing partnership with PLOS and your article meets the relevant criteria, all or part of your publication costs will be covered. Please make sure your user information is up-to-date by logging into Editorial Manager at Editorial Manager® and clicking the ‘Update My Information' link at the top of the page. For questions related to billing, please contact billing support.

If your institution or institutions have a press office, please notify them about your upcoming paper to help maximize its impact. If they’ll be preparing press materials, please inform our press team as soon as possible -- no later than 48 hours after receiving the formal acceptance. Your manuscript will remain under strict press embargo until 2 pm Eastern Time on the date of publication. For more information, please contact onepress@plos.org.

Kind regards,

Miloud Chakit, PhD

Academic Editor

PLOS One

Additional Editor Comments (optional):

Upon editorial assessment of your manuscript and reviewers' reports. All the reviewers' comments have been adequately addressed. I am pleased to inform you that your manuscript is now ready for publication in its present form.

Reviewers' comments:

# Editor report: Acceptance letter

**DOI:** 10.1371/journal.pone.0362368.r006

**Authors:** Miloud Chakit

PONE-D-25-43446R2

PLOS One

Dear Dr. Jonathan,

I'm pleased to inform you that your manuscript has been deemed suitable for publication in PLOS One. Congratulations! Your manuscript is now being handed over to our production team.

At this stage, our production department will prepare your paper for publication. This includes ensuring the following:

* All references, tables, and figures are properly cited

* All relevant supporting information is included in the manuscript submission,

* There are no issues that prevent the paper from being properly typeset

You will receive further instructions from the production team, including instructions on how to review your proof when it is ready. Please keep in mind that we are working through a large volume of accepted articles, so please give us a few days to review your paper and let you know the next and final steps.

Lastly, if your institution or institutions have a press office, please let them know about your upcoming paper now to help maximize its impact. If they'll be preparing press materials, please inform our press team within the next 48 hours. Your manuscript will remain under strict press embargo until 2 pm Eastern Time on the date of publication. For more information, please contact onepress@plos.org.

You will receive an invoice from PLOS for your publication fee after your manuscript has reached the completed accept phase. If you receive an email requesting payment before acceptance or for any other service, this may be a phishing scheme. Learn how to identify phishing emails and protect your accounts at https://explore.plos.org/phishing.

If we can help with anything else, please email us at customercare@plos.org.

Thank you for submitting your work to PLOS One and supporting open access.

Kind regards,

PLOS One Editorial Office Staff

on behalf of

Pr. Miloud Chakit

Academic Editor

PLOS One
